# The endoderm indirectly influences morphogenetic movements of the zebrafish head kidney through the posterior cardinal vein and VegfC

**DOI:** 10.1038/srep30677

**Published:** 2016-08-01

**Authors:** Chih-Wei Chou, Hsiao-Chu Hsu, May-su You, Jamie Lin, Yi-Wen Liu

**Affiliations:** 1 Department of Life Science, Tunghai University, Taichung, Taiwan; 2 Institute of Molecular and Genomic Medicine, National Health Research Institutes, Zhunan, Taiwan

## Abstract

Integration of blood vessels and organ primordia determines organ shape and function. The head kidney in the zebrafish interacts with the dorsal aorta (DA) and the posterior cardinal vein (PCV) to achieve glomerular filtration and definitive hematopoiesis, respectively. How the head kidney co-develops with both the axial artery and vein remains unclear. We found that in endodermless *sox32*-deficient embryos, the head kidney associated with the PCV but not the DA. Disrupted convergent migration of the PCV and the head kidney in *sox32*-deficient embryos was rescued in a highly coordinated fashion through the restoration of endodermal cells. Moreover, grafted endodermal cells abutted the host PCV endothelium in the transplantation assay. Interestingly, the severely-disrupted head kidney convergence in the *sox32-*deficient embryo was suppressed by both the *cloche* mutation and the knockdown of endothelial genes, indicating that an interaction between the endoderm and the PCV restricts the migration of the head kidney. Furthermore, knockdown of either *vegfC* or its receptor *vegfr3* suppressed the head kidney convergence defect in endodermless embryos and perturbed the head kidney-PCV association in wild-type embryos. Our findings thus underscore a role for PCV and VegfC in patterning the head kidney prior to organ assembly and function.

The blood vasculature and its microenvironment play essential roles in shaping organ morphology and function^1^,^2^,^3^. However, how the different types of vasculature and their microenvironments signal to organ tissues remains poorly understood. Genetic analyses revealed a repertoire of early morphogenetic signals that regulate the patterning of organ primordia and axial vessels, suggesting highly parallel developmental processes^4^,^5^,^6^. While defects in both organ and vascular formation have been described, it remains unclear whether and how vessel-organ interactions are perturbed in each morphogenetic mutant or morphant. If the vessel-organ interaction persisted, one would expect a local microenvironment that dominated the interplay between endothelial and organ cells. Therefore, early morphogenetic mutants and morphants may offer valuable platforms for us to dissect the local endothelial microenvironments that pattern various aspects of organ development.

The head kidney in the zebrafish is an excellent model for exploring how endothelium-derived signals shape the developing organ. Organogenesis of the head kidney, which is composed of the pronephric glomerulus (PG) and the interrenal tissue (IR), involves the timely midline convergence of primordial cells prior to their interaction with the dorsal aorta (DA)^7^,^8^. The pronephric kidney in the zebrafish embryo is a two-nephron structure connecting to bilateral kidney tubules and ducts^9^,^10^. The developing nephrons secrete angiogenic factors, thus allowing angiogenic vessels sprouting from the DA to enter the kidney and form the glomerular capillary loops^11^,^12^. The interrenal gland is the teleostean counterpart of the mammalian adrenal gland and is composed of steroidogenic interrenal and chromaffin cells^13^. Bilateral IR clusters are specified within and segregated from the pronephric nephrons, and they subsequently coalesce and relocalize to the space juxtaposing the DA and the right branch of the paired PCV^14^. The fused IR then extends ventral to the DA and across the midline, where it interacts with the interrenal vessel (IRV) that is derived from the DA^15^. These studies indicate a close interaction between the DA and the head kidney organs during organ assembly. The head kidney sits at the rostral end of the DA/PCV joint where hematopoietic stem cells emerge^16^,^17^, and is juxtaposed between the DA and the radix of the paired PCV branches (Fig. S1). The homing of the hematopoietic precursors from the caudal hematopoietic tissue through the PCV renders the pronephros one of the definitive sites of hematopoiesis. It remains unclear how the PCV endothelium integrates with the developing head kidney during embryogenesis. In the *one-eye pinhead (oep*) and *squint (sqt*) mutants in which the Nodal signalling is defective, the midline convergence of the head kidney and the formation of the PCV are both severely defective^4^,^18^,^19^. However, assembly and angiogenesis of the DA are unperturbed in *oep. oep* and *sqt* are required for the expression of *sox32,* which controls the endodermal fate^20^,^21^. Hence, the head kidney and trunk vasculature phenotypes in Nodal mutants implicate a morphogenetic relationship among the endoderm, the PCV and the kidney.

The endoderm is believed to orchestrate midline convergence of multiple mesodermal tissues. In the endodermless *sox32 (casanova, cas*) mutant, mesodermal organs including the heart, vasculature, blood and kidney demonstrate convergence defects^22^. However, final assembly of the DA and the PCV at the lower trunk is generally unperturbed in *cas* mutants. The migration of venous angioblasts is significantly delayed, indicating a role for the endoderm in the temporal regulation of venous tube formation^23^. While the precursors of pronephric and vascular cells are associated during both midline convergence, it remains unclear whether the interplay is altered in endodermless embryos. We therefore analysed the morphology of the PG, the IR, and their adjacent midtrunk vessels in the *cas* mutant and morphant. Despite normal DA formation in endodermless embryos, neither the PG nor IR tissues migrated toward the midline. The endothelium that constitutes the paired branches of the PCV was defective in central migration and remained associated with the bilateral PG and IR. We showed that the defective PG, IR and PCV morphogenesis at the midtrunk in *cas* mutants was not due to perturbed laterality but to a loss of endoderm. Surprisingly, while the endothelium-free *cloche (clo*) mutant also demonstrated a fusion defect of the bilateral PG and IR, this mutation significantly suppressed the severe convergence phenotype in endodermless embryos. A knockdown of endothelial genes was performed in the endodermless embryos to verify that the suppressing effect of *clo* was due to a loss of endothelium in the head kidney region. We further show that this novel phenomenon was due to a persistent interaction between the primordial cells of the head kidney and the PCV at least in part through VegfC/Vegfr3 (Flt4) signalling. Moreover, VegfC/Flt4 signalling was required for the positioning of the head kidney at the rostral end of the DA-PCV joint. We therefore underscored a novel role for VegfC in embryonic kidney development.

## Results

### Convergence and assembly of PG and IR tissues is disrupted in the *cas* mutant

**Figure 1:**
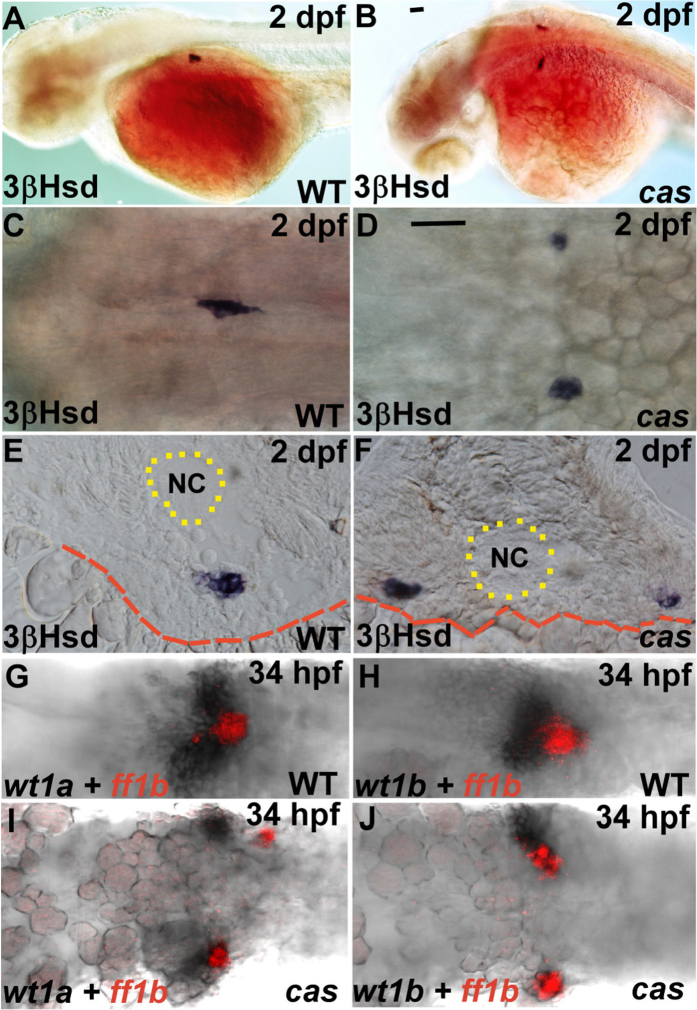
The phenotypes of the PG and the IR in the *cas* mutant and its wild-type sibling. (**A–F**) The steroidogenic IR detected by whole-mount 3β-Hsd staining at 2 dpf in the *cas* mutant (**B,D,F**) and its wild-type sibling (**A,C,E**). Dorsolateral (**A,B**) and dorsal (**C,D**) views are oriented with the anterior toward the left. (**E,F**) Cryosections with dorsal oriented toward the top. (**G–J**) Expression of *ff1b* and *wt1a* (**G,I**) or *wt1b* (**H,J**) were detected simultaneously in the *cas* mutant (**I,J**) and its wild-type sibling (**G,H**). The boundaries of the yolk and the notochord (NC) are highlighted by red dashed and dotted lines, respectively. Scale bar, 50 μm.

To examine whether and how head kidney morphogenesis is disrupted in the endodermless embryo, we first assessed the functional differentiation of the IR, the last-differentiated segment of the kidney field, in the *cas* embryo and its wild-type sibling. Whole-mount 3β-hydroxysteroid dehydrogenase (3β-Hsd) enzymatic staining that detects steroidogenic cells indicated that morphogenesis but not differentiation of the IR was disrupted in the *cas* mutant, where the bilateral IRs failed to migrate toward the midline (Fig. 1A–F). This finding suggested that Sox32-mediated endoderm formation is required for the convergence but not differentiation of the head kidney.

The PG and the IR develop in a highly parallel manner during development^7^. To check whether this parallel pattern of development persists in the *cas* mutant, the expression of *ff1b (nr5a1a*), which marks the ontogeny of the IR, was detected together with that of *wt1a* (Fig. 1G,I) and *wt1b* (Fig. 1H,J). *wt1a* and *wt1b* exhibit partially-overlapping expression patterns in the embryonic kidney field and control distinct steps of pronephric development^24^,^25^,^26^. The results in Fig. 1(G–J) displayed that both *wt1a* and *wt1b* were expressed in the proximity of *ff1b*. This association was unperturbed in the *cas* mutant, indicating that convergent migration but not the segmentation and differentiation of the kidney field was disrupted in the *cas* mutant.

### Morphology of the PCV but not the DA in the vicinity of the head kidney is perturbed in the *sox32* morphant

**Figure 2:**
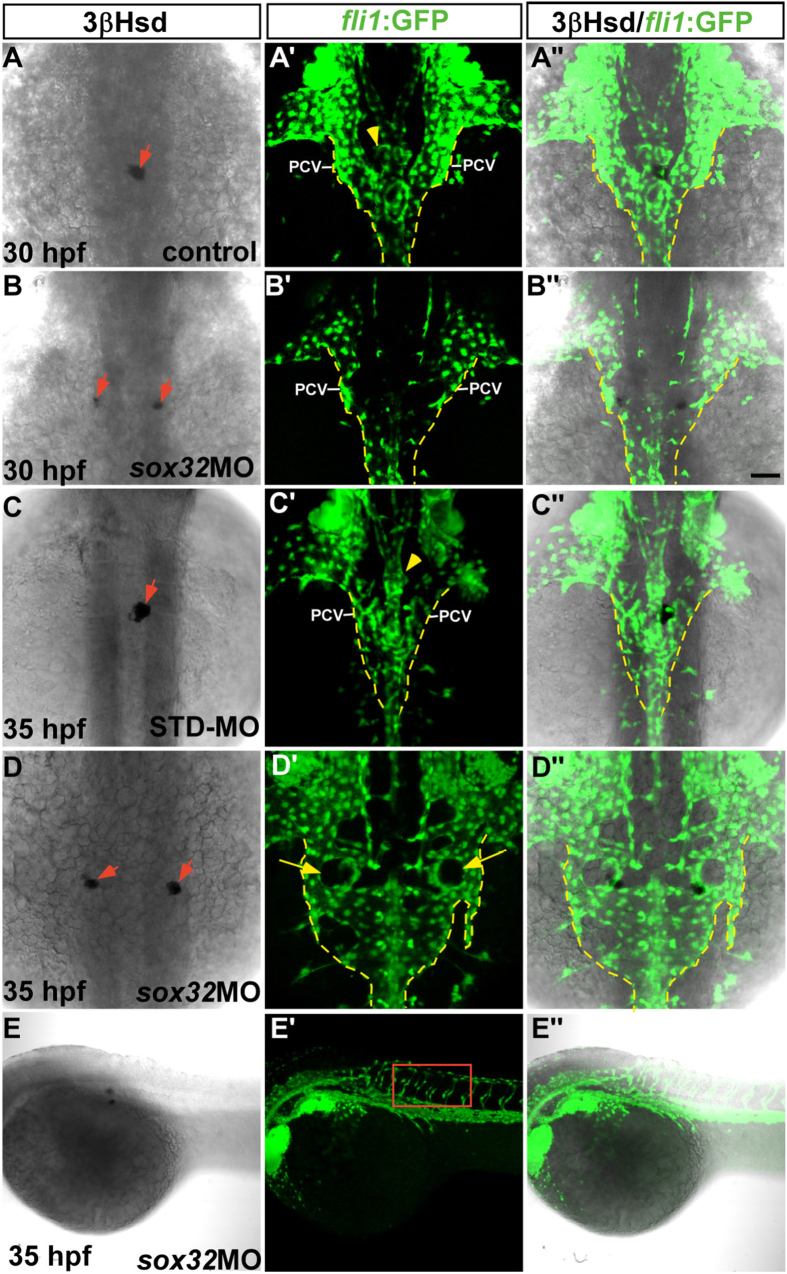
The morphology of the axial vasculature in the vicinity of the kidney and the IR in the *sox32* morphant. 3β-Hsd activity staining was performed on the *sox32*MO-injected *Tg(fli1:EGFP)*^*y1*^ embryos (**B–B”,D–D”,E–E”**) and the uninjected (**A–A”**) or STD-MO-injected (**C–C”**) controls. All panels except (**E–E”**) are dorsal views with anterior oriented toward the top, while (**E–E”**) are dorsolateral views with anterior toward the left. The outlined area in **E**’ highlights the normal growth of the intersegmental vessels in the *sox32* morphant. Yellow broken lines demarcate lateral boundaries of the PCV. Red arrows indicate IRs. Yellow arrowheads indicate PGs. Yellow arrows denote the pronephric tissues deficient in angiogenesis. Scale bar, 50 μm.

The head kidney phenotype in the *cas* mutant was reminiscent of that in the endothelium-free mutant *clo*^14^. To understand whether the defective head kidney convergence in the *cas* mutant was related to an abnormal vascular phenotype, we assessed the vascular pattern in the vicinity of the PG and IR in the *sox32* morphant. Phenocopying the *cas* mutant, the midline convergence of bilateral IRs was severely disrupted in the *sox32*morpholino (MO) injected *Tg(fli1:EGFP)*^*y1*^ embryo (Fig. 2B–B”,D–D”), whose vascular structure was delineated by GFP. Consistent with the results of Jin *et al*.^23^, there was no evident disruption of axial vessels at the lower trunk region of *sox32*MO-injected *Tg(fli1:EGFP)*^*y1*^ embryos (Fig. 2E–E”). The normal sprouting of intersegmental vessels from the DA in the *sox32* morphant further indicated that the central assembly and formation of the axial artery in the *sox32*-deficient embryo was unaffected. However, the paired PCV branches at the midtrunk of the *sox32* morphant were morphologically abnormal at 35 hpf (Fig. 2D’–D”). Their boundaries were more laterally displaced than those in the control embryo (Fig. 2C’–C”), indicating a defective convergence of venous cells. This dramatic difference in PCV structures between the *sox32* morphant and the control was not obvious at 30 hpf (Fig. 2A–A”,B–B”), suggesting that the disrupted convergence of venous cells occurred between 30 to 35 dpf. While the assembly of the PCV but not the DA in the vicinity of the head kidney was perturbed, the head kidney in the *sox32* morphant was associated with the PCV but not the DA (Fig. 2B”,D”). In the normal embryo, angiogenic sprouts from the DA invade the PG and form a capillary loop immediately rostral to the 3β-Hsd-positive interrenal region (Figs 2A–A”,C–C” and S2, yellow arrowheads). PG angiogenesis was absent in the *sox32* morphant (Figs 2D–D” and S2, yellow arrows), which was consistent with a disrupted association of the head kidney with the DA. It is therefore of interest to explore whether the convergence defect of the head kidney in the *sox32*-deficient embryo was related to the defective assembly of PCV structures at the midtrunk.

### The phenotype of the head kidney in the *cas* mutant is not due to perturbed laterality

**Figure 3:**
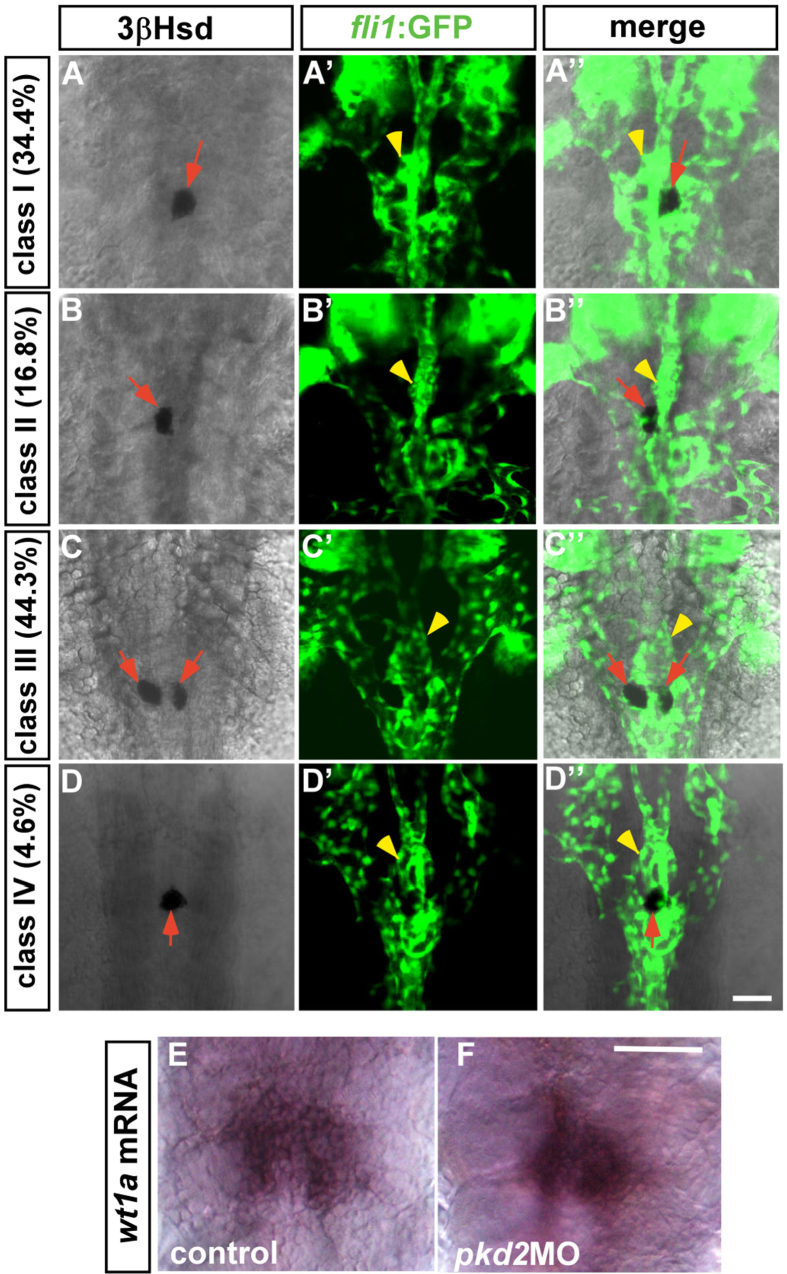
The phenotypes of the PG and the IR in the *pkd2* morphant at 2 dpf. (**A–D,A’–D’,A”–D”**) The *Tg(fli1:EGFP)*^*y1*^ embryos injected with the *pkd2* MO displayed four classes of phenotypes at 2 dpf, which could be categorized according to various types of interrenal morphology. Class I: single IR cluster to the right of the midline (**A**–**A”**); Class II: single IR cluster to the left of the midline (**B**–**B”**); Class III: bilateral IR clusters immediately adjacent to the midline (**C**–**C”**); Class IV: single IR cluster at the midline (**D**–**D”**). There appeared to be no defects in glomerular angiogenesis (yellow arrowheads) in all classes. Red arrows indicate IRs. (**E,F**) Expression of *wt1a* was detected in the *pkd2* morphant as well as the control embryo at 2 dpf. The images of the control embryo and the *pkd2* morphant are representative of 23 and 13 samples, respectively. Scale bar, 50 μm.

Embryos lacking *sox32* function fail to develop normal dorsal forerunner cells and their derivative Kupffer’s vesicle, a ciliated organ that determines left-right asymmetry^22^,^27^. This implied the possibility that the head kidney phenotype in the *cas* mutant was due to a general disruption in organ asymmetry other than the absence of endoderm. To address this, we measured the IR phenotype in embryos deficient in the expression of *pkd2,* which is regulated downstream of Sox32 and required for specifying left-right asymmetry but not endoderm formation^28^,^29^. While the *pkd2*MO-injected *Tg(fli1:EGFP)*^*y1*^ embryo displayed a curly tail phenotype (Fig. S3) that phenocopied the *curly up (pkd2*) mutant^28^, the left-right asymmetry of the IR was randomized upon the down-regulation of *pkd2* (Fig. 3). Among the four classes of IR phenotypes in the *pkd2* morphant, class III appeared to display a mild fusion defect, which could be due to a duplication of IR tissues. Duplication of lateralized organs has been reported in the *pkd2* mutant^28^. Meanwhile, the *pkd2* morphant did not display a convergence phenotype for the PCV adjacent to the IR (Fig. 3A’–D’,A”–D”). It is noteworthy that angiogenesis of the PG (yellow arrowheads in Fig. 3A’–D’,A”–D”) as well as the fusion of bilateral PG (Fig. 3F) were unperturbed in the *pkd2* morphant. This indicates that the head kidney phenotypes in the *cas* mutant were specifically due to the loss of endoderm and not disrupted left-right asymmetry.

### Defective convergence of the head kidney and the PCV in the *sox32* morphant is rescued by the simultaneous knockdown of *odd-skipped-related 1 (osr1*)

**Figure 4:**
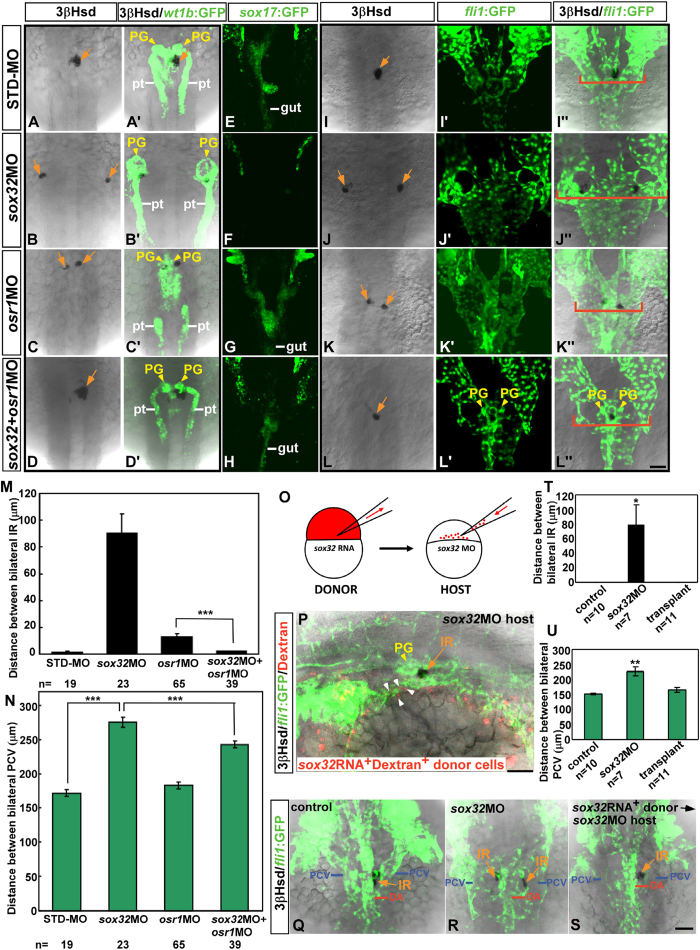
Reintroduction of endodermal cells into the endodermless embryo rescues defective migration of the head kidney and the PCV. (**A**–**N**) The inhibition of *osr1* expression led to a rescue of renal, interrenal and PCV phenotypes in the *sox32* morphant. Dorsal views of *Tg(wt1b:GFP)* (**A**–**D,A’**–**D’**), *Tg(sox17:EGFP)*^*s870*^(**E**–**H**) and *Tg(fli1:EGFP)*^*y1*^(**I**–**L,I’**–**L’,I”**–**L”**) embryos injected with *sox32*MO, *osr1*MO, *sox32/osr1* double-MOs or STD-MO, respectively, and harvested at either 34 hpf (**A**–**D,A’**–**D’,E**–**H**) or 36 hpf (**I**–**L,I’**–**L’,I”**–**L”**) for labelling steroidogenic IR (orange arrows) by 3β-Hsd activity staining. PG and pronephric tubules (pt) were detected by GFP expression in *Tg(wt1b:GFP)*, while the gut tube was detected by GFP in *Tg(sox17:EGFP)*^*s870*^. The embryos are oriented with anterior to the top. Distances between bilateral IRs and the outer edges of the PCVs at the level of the IR as described in (**I**–**L,I’**–**L’**) are quantified in (**M**,**N**). When the *osr1* MO was co-injected with the *sox32* MO, the convergence defects of bilateral IRs and PCVs were significantly alleviated. Red brackets mark lateral boundaries of the PCV branches at the level of the IR. (**O**–**U**) Transplantation of *sox32-*expressing cells into the *sox32* morphant rescued the defective migration of bilateral PCVs and IRs. (**O**) Schematic of the transplantation approach using Sox32 to target donor cells to the endoderm. (**P**) The lateral view of a representative recipient (n = 11/12) shows that grafted *sox32*RNA^+^Dextran^+^ cells were associated with the PCV endothelium. The *sox32*RNA^+^Dextran^+^ cells near the PCV are marked by white arrowheads. The embryo is oriented with anterior to the left. Dorsal views of the IR and the PCV in the control, the *sox32* morphant and the recipient are shown in (**Q**–**S**), and the distance between bilateral IRs and PCVs are quantified in (**T**,**U**) respectively. *P < 0.05; **P < 0.005; ***P < 0.001 (Student’s t-test). Scale bar, 50 μm.

The defective convergence and assembly of the PCV and the head kidney in the *sox32*-deficient embryo was alleviated when the lost endoderm was rescued by the simultaneous knockdown of *osr1* (Fig. 4A–N). *osr1* limits early endodermal differentiation from the mesendoderm, and its knockdown causes endodermal expansion that in turn alters the balance between renal vs. vascular development^30^. The expanded endoderm in the *osr1* morphant causes a loss of proximal pronephric tubules and an increase in angioblasts. This imbalance of mesodermal differentiation is alleviated by a reduction of endoderm through the co-injection of *sox32*MO. In contrast, in this study the *osr1*MO was utilized to rescue the loss of endoderm in the *sox32* morphant to test whether the endoderm is essential for patterning the convergence of the head kidney and PCV cells at the midtrunk.

To explore how the genetic interaction between *sox32* and *osr1* affected the convergence of PG and IR primordia, *sox32*MO and *osr1*MO were injected individually or together into *Tg(wt1b:GFP)* embryos (Fig. 4A–D,A’–D’). 3β-Hsd activity staining in *Tg(wt1b:GFP)* embryos whose glomerular podocytes, pronephric tubules and proximal pronephric ducts were marked by GFP^26^,^31^ revealed the relative distribution of both the PG and the IR. In all of the treatments, the PG and the IR remained associated, suggesting that the parallel development of the PG and the IR persisted when the Sox32-Osr1 genetic interaction was disrupted. Consistent with the previous finding by^30^, proximal pronephric tubules were lost and glomerular podocytes were retained in the *osr1* morphant (83.9%, n = 31; a representative embryo is shown in Fig. 4C,C’). Moreover, specification of the IR was not disrupted in the *osr1* morphant (Fig. 4C,C’; 100%, n = 31), which agrees with the fact that the IR is derived from the primordial PG^7^. While the loss of proximal pronephric tubules in the *osr1* morphant was rescued by co-injected *sox32*MO (100%, n = 14; a representative embryo is shown in Fig. 4D,D’), the severe convergence defect of the head kidney in the *sox32* morphant was alleviated by co-injected *osr1*MO. Consistently, immunohistochemistry (IHC) using the anti-Na^+^/K^+^-ATPase antibody detected a loss of proximal convoluted tubules (PCTs) in the *osr1* morphant at 53 hpf, which was rescued as the *sox32*MO was co-injected (Fig. S4). On the other hand, the average distance between the bilateral outer boundaries of the PCTs at 34 hpf was significantly larger in the *sox32* morphant (193.6 ± 7.7 μm, n = 57) than in either the STD-MO-injected (104.8 ± 2.3 μm, n = 21) or *sox32*MO/*osr1*MO co-injected *Tg(wt1b:GFP)* embryos (133.0 ± 5.9 μm, n = 33). These results thus indicate that the genetic interaction between *sox32* and *osr1* ensures not only the fate specification but also the convergence of the developing kidney prior to its segmental differentiation.

The effect of *sox32*MO and *osr1*MO on endodermal tissue was validated in the endoderm-specific reporter line *Tg(sox17:EGFP)*^*s870*^ (Fig. 4E–H). The endodermal tissue was absent at the midtrunk of the *sox32* morphant (Fig. 4F) and was expanded at the midtrunk of the *osr1* morphant (Fig. 4G). The embryo co-injected with *sox32*MO and *osr1*MO displayed a hypomorphic yet evident gut tube structure, implicating a partial rescue of endoderm. These results shown in the *Tg(sox17:EGFP)*^*s870*^ embryo were further validated by an *in situ* hybridization (ISH) analysis of the *forkhead box A2 (foxa2*) expression (Fig. S5) which marks the early endoderm^20^, indicating that the genetic interaction between *sox32* and *osr1* regulates the midline convergence of the head kidney by modulating endoderm formation.

To analyse whether the defective convergence of head kidney-associated vascular cells in the *sox32* morphant could also be rescued by co-injected *osr1*MO, 3β-Hsd-activity staining was performed on *Tg(fli1:EGFP)*^*y1*^ embryos injected with *sox32*MO and *osr1*MO either individually or together (Fig. 4I–L,I’–L’,I”–L”). The severely defective convergence of the PCV in the *sox32* morphant, as estimated by the distance between the bilateral PCV at the level of the IR (bracketed in Fig. 4I”–L”), was partially yet significantly rescued in the embryo co-injected with *sox32*MO and *osr1*MO. No significant variation was found between the STD-MO injected control and the *osr1* morphant embryos (Fig. 4N). The deficient glomerular angiogenesis in the *sox32* morphant was not rescued in the co-injected embryo (Fig. 4L’,L”), suggesting that the partial rescue of the endoderm in *sox32*MO/*osr1*MO co-injected embryos was insufficient to restore the interaction between the glomerular podocytes and the arterial endothelium.

Interestingly, while the bilateral IR primordia were associated with the PG and the PCV in the *sox32* morphant, 65% of *sox32*MO/*osr1*MO co-injected *Tg(fli1:EGFP)*^*y1*^embryos (n = 39) displayed a single interrenal cluster at the midline, suggesting a strong restorative effect for the convergence of IR primordia (Fig. 4L–L”). Nevertheless, the *osr1*MO injection led to a mild convergence defect for the bilateral IRs (Fig. 4K–K”,M) that might result from defective endodermal structures (Fig. 4G). The average distance between the bilateral IRs in *sox32*MO/*osr1*MO co-injected embryos was significantly lower than that in either the *sox32* or *osr1* morphants (Fig. 4M), indicating that balanced endoderm formation is essential for head kidney convergence. Moreover, these results suggest that head kidney convergence and its associated PCV vasculature could be regulated by the endoderm in a highly coordinated manner.

### Grafted endodermal cells rescued defective migration of bilateral IRs and PCVs in the Sox32-deficient embryo

To further verify whether endodermal cells modulate the migration of bilateral head kidney and PCV tissues during development, a transplantation assay was performed by grafting Sox32-overexpressing cells into the *sox32* morphant (Fig. 4O–U). Donor cells from embryos co-injected with *sox32* mRNA and rhodamine-dextran were placed along the blastoderm margin of the *sox32*MO-injected *Tg(fli1:EGFP)*^*y1*^ (Fig. 4O). Notably, the recipients harbouring transplanted cells at the midtrunk (n = 11/12) consistently displayed an association between the transplanted cells and the host venous vasculature (Figs 4P and S6), indicating a strong reciprocal interaction between endodermal and venous cells. Meanwhile, the transplanted cells formed a gut tube-like structure at the posterior trunk region (Fig. S7), suggesting that they were targeted to the endoderm. The recipient embryos with grafted donor cells abutting the PCV endothelium displayed normal convergence of bilateral IRs as well as PCV structures (Fig. 4Q–U). The grafted cells were associated with the PCV endothelium but not the head kidney, supporting that the endoderm modulates the convergence of the head kidney through patterning the midtrunk venous structures.

### Loss of endothelium suppresses the convergence defect of the IR in endodermless embryos

**Figure 5:**
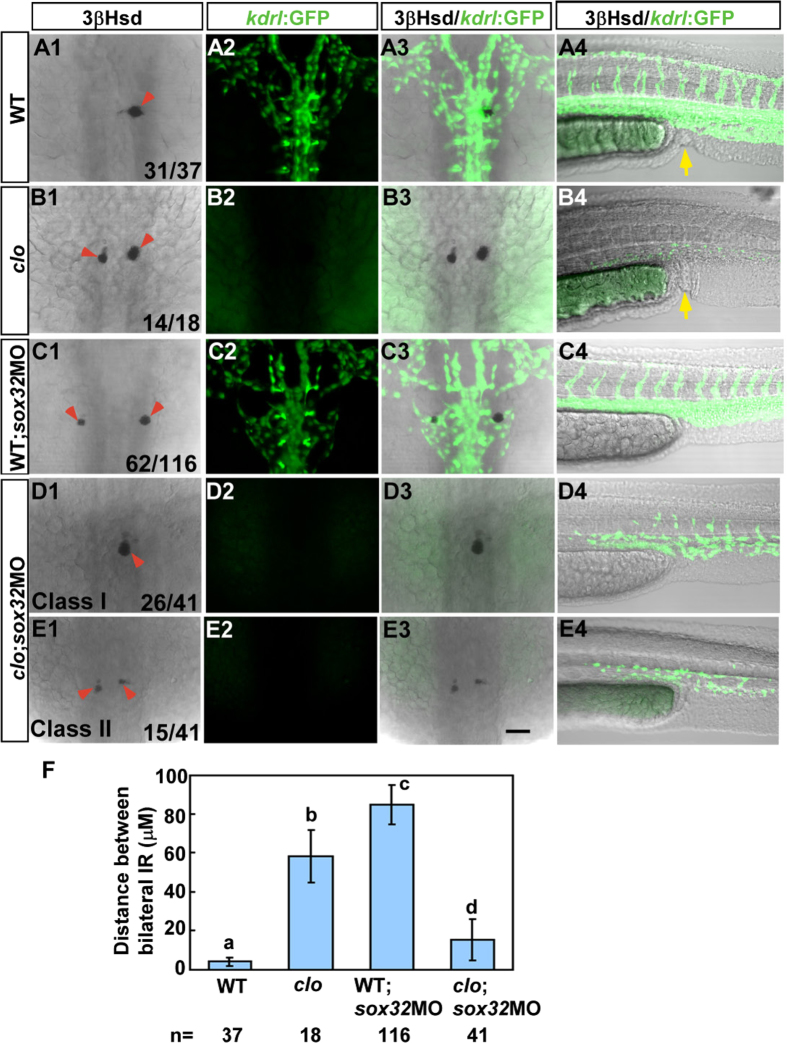
The defective interrenal migration in the *sox32* morphant is suppressed by the *clo*^*m39*^ mutation. The *sox32*MO was injected into the *Tg(kdrl:EGFP)*^*s843*^*; clo*^*m39*^ mutant (**D1**–**D4,E1**–**E4**) and its wild-type sibling (**C1**–**C4**) to inhibit endoderm formation, as verified by the absence of anal openings (**C4**–**E4**). By contrast, the *clo*^*m39*^ mutant (**B1**–**B4**) and its wild-type sibling (**A1**–**A4**) showed normal formation of anal openings (yellow arrows in **A4 and B4**). All images are single confocal sections showing the IR as detected by 3β-Hsd staining (**A1**–**E1,A3**–**E3**) and the vascular pattern at the midtrunk (**A2**–**E2,A3**–**E3**) and the lower trunk (**A4**–**E4**) revealed by GFP in *Tg(kdrl:EGFP)*^*s843*^ at 34 hpf. In *clo*^*m39*^, the endothelial fluorescence was absent at the midtrunk and disrupted at the lower trunk. The severe disruption of interrenal migration in the *sox32* morphant was rescued when the endothelium was simultaneously deleted by the *clo*^*m39*^ mutation, as revealed in both class I (**D1**–**D3**) and class II (**E1**–**E3**) double loss-of-function embryos. The effects of various treatments on the convergence of bilateral IRs are quantified in (**F**). Histograms with different letters above them are significantly different (ANOVA and Duncan’s multiple test, *P* < 0.05). Scale bar, 50 μm.

To explore whether the morphogenetic movements of the head kidney are primarily influenced by the adjacent vasculature patterned by the endoderm or whether the migrations of the head kidney and the PCV cells are co-regulated by the endoderm, we analysed the morphology of the IR, the last-differentiated segment of the kidney field, in the absence of both endoderm and endothelium (Fig. 5). The *sox32*MO was injected into the mutant embryos and wild-type siblings of a *clo*^*m39*^ strain that had been crossed to *Tg(kdrl:EGFP)*^*s843*^ line to monitor endothelial development. Consistent with our previous study^14^, defective fusion of bilateral IRs was detected in the *Tg(kdrl:EGFP)*^*s843*^*; clo*^*m39*^ embryo (Fig. 5B1). In contrast, midline convergence of bilateral IRs was much more severely disrupted in the *sox32*MO-injected wild-type sibling of *Tg(kdrl:EGFP)*^*s843*^*; clo*^*m39*^ (Fig. 5C1), hinting at an earlier role for the endoderm than for the endothelium in the convergence of the head kidney. The endoderm might regulate the parallel migrations of the endothelial and pronephric cells before the axial vasculature modulates the final fusion and assembly of the bilateral head kidney at the midline. Therefore, a double loss-of-function of *sox32* and *clo* would result in severe convergence defects for the bilateral head kidney. However, the interrenal convergence defect in the absence of the endoderm was fully suppressed in 66% of the *sox32*/*clo* double loss-of-function embryos (Fig. 5D1, 2, 3), while the rest displayed a fusion phenotype similar to *clo*^*m39*^(Fig. 5E1). The suppression of the interrenal convergence phenotype in the *sox32*MO-injected *Tg(kdrl:EGFP)*^*s843*^*; clo*^*m39*^ embryo was not due to an alleviation of endodermal deficiency, as the loss of the cloaca (terminal segment of the primitive gut) was verified as a marker of the endodermless state in all *sox32*MO-injected embryos (Fig. 5C4–E4). *clo*^*m39*^ and its wild-type siblings demonstrated normal cloaca formation (Fig. 5A4; yellow arrows). This suggests that the severely disrupted interrenal convergence in *sox32*-deficient embryos was secondary to the defective assembly of the midtrunk vasculature. Therefore, the endoderm might pattern the PCV and subsequently modulate the migratory activity of the undifferentiated intermediate mesoderm. Indeed, the convergence and morphology of the bilateral PCV was disrupted at the midtrunk (Fig. 5C2) but not the lower trunk (Fig. 5C4) of the *sox32*MO-injected wild-type siblings of *Tg(kdrl:EGFP)*^*s843*^*; clo*^*m39*^, consistent with the phenotype revealed in *sox32*MO-injected *Tg(fli1:EGFP)*^*y1*^ embryos (Fig. 2D’,E’). It is therefore necessary to examine whether the morphogenesis of the whole kidney field is influenced by an interaction between the endoderm and the endothelium.

### Convergence defects of the kidney in the *sox32* morphant are partially rescued by a knockdown of either *ets variant 2 (etv2*) or *scl*

**Figure 6:**
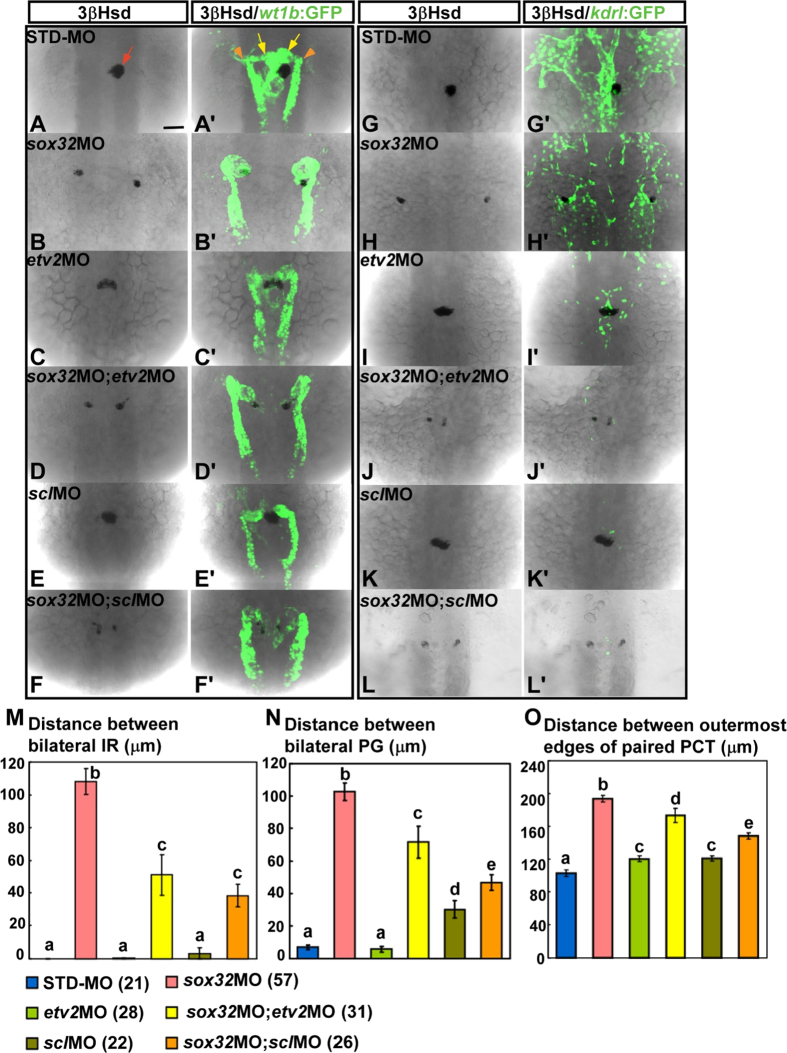
The defective midline convergence of the PG and the IR in the *sox32* morphant at 34 hpf is suppressed by both *etv2*MO and *scl*MO. *sox32*MO, *etv2*MO, *scl*MO, *sox32/etv2* double-MOs, *sox32/scl* double-MOs and STD-MO were injected into either *Tg(wt1b:GFP)* (**A**–**F,A’**–**F’**) or *Tg(kdrl:EGFP)*^*s843*^ (**G**–**L,G’**–**L**’) embryos. The injected embryos were subjected to 3β-Hsd staining and analysed for the effects of MOs on the morphology of the kidney delineated by *wt1b:GFP* (**A’**–**F’**), the axial vasculature by *kdrl:EGFP* (**G’**–**L’**), and the IR by 3β-Hsd (**A**–**L**). Distances between bilateral IRs, bilateral glomeruli and the outermost edges of paired PCTs in injected *Tg(wt1b:GFP)* embryos are quantified in (**M**–**O**), respectively, with the number of samples indicated in parentheses. The IR, the glomeruli and the PCT are denoted by red arrow, yellow arrows and orange arrowheads, respectively. Histograms with different letters above them are significantly different (ANOVA and Duncan’s multiple test, *P* < 0.05). Scale bar, 50 μm.

To examine whether and how the severely defective convergence of kidney structures in endodermless embryos could be alleviated by a simultaneous deletion of the endothelium, MO against either *etv2* or *scl* was co-injected with *sox32*MO into the *Tg(wt1b:GFP)* embryo (Fig. 6A–F,A’–F’). The effects of the MO injections on the midtrunk vasculature were tested using the *Tg(kdrl:EGFP)*^*s843*^ embryo (Fig. 6G–L,G’–L’). *etv2* and *scl* function downstream of *clo* and play different roles in the development of the hemangioblast lineage^32^,^33^. While *etv2* specifically mediates endothelial development, *scl* is required for both hematopoiesis and vasculogenesis. Consistent with the previous findings, both *etv2*MO and *scl*MO led to a severe reduction of endothelial cells at the midtrunk (Fig. 6I’,K’) that phenocopied the *clo*^*m39*^ mutant (Fig. 5B2). Unlike the *clo* mutation, knockdown of either *etv2* or *scl* did not significantly affect the fusion of bilateral IRs (Fig. 6C,E,M), which could be due to an incomplete depletion of endothelium at the midtrunk. However, both *etv2*MO and *scl*MO significantly rescued the defective convergence of bilateral PGs and IRs in the *sox32* morphant (Fig. 6D–D’,F–F’) in which endoderm formation was inhibited (Fig. S8). Interestingly, the rescue effects of either *etv2*MO or *scl*MO in midline convergence were more evident for the bilateral IR (Fig. 6M) than for the PG (Fig. 6N), which was consistent with the fact that the fusion of the bilateral IR occurs earlier than that of the PG. By contrast, the convergence of paired PCTs in the *sox32* morphant was mildly rescued by either *etv2*MO or *scl*MO (Fig. 6O). This suggested that the rescue effects of *etv2*MO or *scl*MO on midline convergence were evident for the terminally differentiated segments of the kidney that assemble at the midline. The original positioning of the undifferentiated bilateral PG in the endodermless embryo could not be rescued by either *etv2*MO or *scl*MO (Fig. S9), indicating that the head kidney phenotypes in the *sox32*/*etv2* and *sox32*/*scl* double-morphants were not due to an emergence of kidney primordia near the midline. Consistently, the original positioning of the undifferentiated bilateral IR in the endodermless embryo could not be rescued by the *etv2*MO (Fig. S10).

**Figure 7:**
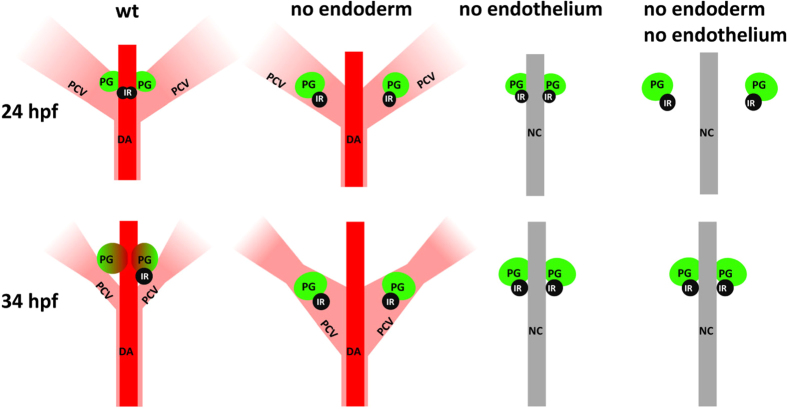
Morphogenetic movements of the PG and the IR are regulated by an interaction between the endoderm and the endothelium. The panels represent dorsal views of 24 and 34 hpf embryos at the midtrunk level oriented with anterior to the top. Morphologies of the DA, the PCV, the PG and the IR are depicted according to the phenotypes in wild-type (wt), endodermless, and endothelium-free embryos, as well as embryos deficient in both endoderm and endothelium. NC, notochord.

Summarized from the results above, an association between the head kidney and the PCV was revealed in endodermless embryos, and both displayed severely disrupted and irreversible convergence defects (Fig. 7). Further depletion of the trunk endothelium in endodermless embryos partially yet significantly restored the convergence of the head kidney, suggesting that the head kidney migration defect is secondary to that of PCV cells. This finding also suggests that although the DA is important for the final assembly and angiogenesis of the head kidney, it is not involved in the earlier convergence movements of head kidney cells.

### The parallel migration of head kidney tissues with the PCV requires VegfC

**Figure 8:**
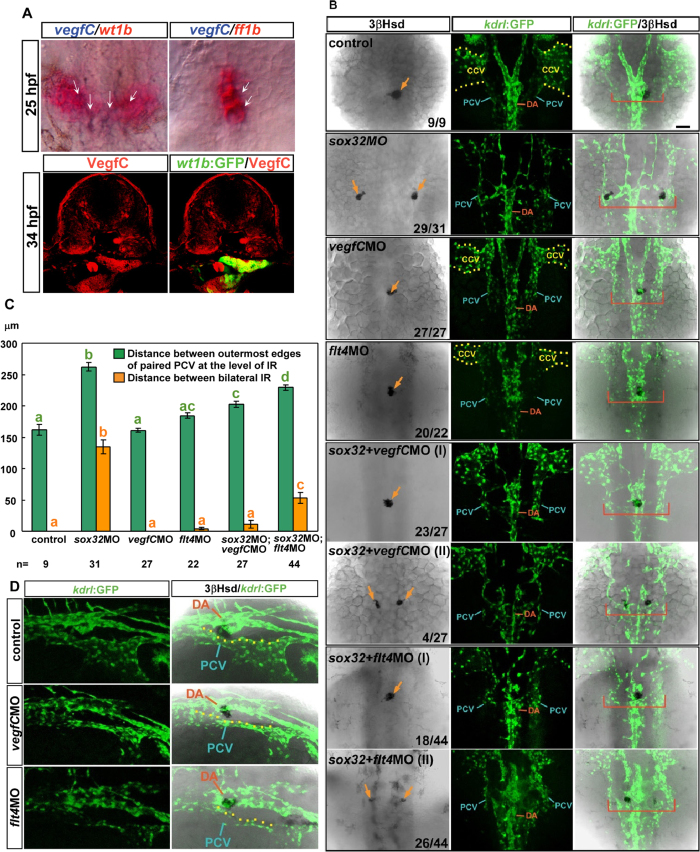
Both *vegfC*MO and *flt4*MO rescue the convergence of the IR in *sox32*-deficient embryos. (**A**) (Upper) Colocalization of *vegfC* with *wt1b* and *ff1b*, respectively, in the 25 hpf embryo by ISH. (Lower) A cross-section of a 34 hpf embryo showing that VegfC protein (red) was enriched in the kidney (delineated by *wt1b:GFP*). (**B**) *sox32*MO, *vegfC*MO, *flt4*MO, *sox32/vegfC* double-MOs, *sox32/flt4* double-MOs were injected into *Tg(kdrl:EGFP)*^*s843*^ embryos. The injected embryos were subjected to 3β-Hsd staining and analysed for the effects of MOs on the morphology of the IR (delineated by the 3β-Hsd activity and marked by orange arrows) and the axial vasculature (delineated by *kdrl:EGFP*). Dorsal views, with anterior to the top. Distances between bilateral IRs and between the bilateral edges of the PCV at the level of the IR (marked by red brackets) are quantified in (**C**). (**D**) Dorsolateral views of the control, *vegfC*MO-injected, and *flt4*MO-injected *Tg(kdrl:EGFP)*^*s843*^ embryos at 34 hpf, with anterior to the left, to show the relative distribution of the DA, PCV and IR. Scale bar, 50 μm.

The developing PCV, the common cardinal vein (CCV), the caudal vein and the intersegmental vein express Flt4 (Vegfr3), which interacts with VegfC during vasculogenesis, angiogenesis and lymphangiogenesis^6^,^34^,^35^. In addition, VegfC expressed in circulating nucleate erythrocytes interacts with Flt4 in the CCV and thus promotes lumen ensheathment^36^. *vegfC* mRNA has also been reported to be expressed in the embryonic kidney^6^. Our ISH analysis revealed a weak yet clear expression of *vegfC* RNA in part of the *wt1b*-expressing PG and *ff1b-*expressing IR domains (Fig. 8A). IHC was also performed to confirm the expression of VegfC in the developing head kidney. However, whether VegfC is involved in pronephric kidney development remains unknown.

To test if VegfC/Flt4 signalling is involved in the parallel migration of the head kidney with the PCV, we analysed the morphology of the axial vasculature and the IR in embryos injected with *vegfC*MO and *flt4*MO, respectively, and in those co-injected with either *vegfC*MO/*sox32*MO or *flt4*MO/*sox32*MO (Fig. 8B,C). Consistent with the previous findings in the *vegfC* mutant^36^, the CCV structure is hypomorphic in the *vegfC* and *flt4* morphants (Fig. 8B). In all *vegfC* morphants (27/27) and in the majority of *flt4* morphants (20/22), a single IR cluster was detected at the midline, indicating the successful convergence of head kidney tissues. However, the converged IR displayed a poor affinity to the PCV compared to controls (Fig. 8D). Successful convergence of the IR was also observed in 85% of the *vegfC*MO/*sox32*MO co-injected and 41% of the *flt4*MO/*sox32*MO co-injected embryos, while the rest of co-injected embryos displayed a milder head kidney convergence phenotype compared to that of the *sox32* morphant. Therefore, both *vegfC*MO and *flt4*MO suppressed the severe convergence defect of the head kidney tissues in the endodermless embryo. The defective convergence of the PCV in the endodermless *sox32* morphant was slightly restored when either *vegfC*MO or *flt4*MO was co-injected (Fig. 8C). However, the morphology of the PCV in the *vegfC*MO/*sox32*MO and *flt4*MO/*sox32*MO morphants was similar to that in the *sox32* morphant (Fig. 8B). In addition, while the widely bilateral IR primordia in the *sox32* morphant were only associated with the PCV endothelium, the converged IR in *vegfC*MO/*sox32*MO or *flt4*MO/*sox32*MO co-injected embryos was associated with the DA. This evidence indicates that VegfC/Flt4 signalling is required for the parallel migration of head kidney tissues with the PCV.

## Discussion

In this study, we used a combination of genetic mutants and the MO knockdown to unravel the interaction among the endoderm, the axial vasculature and the kidney. While the clo^*m39*^ mutant was used to examine the role of the endothelium for the head kidney development, the *clo* gene remains unidentified. Therefore, it is questionable whether the *clo* gene could also be playing a cell-autonomous role in the intermediate mesoderm. This possibility could however be ruled out, as the migration defect of the head kidney in the clo^*m39*^ mutant could be alleviated by the forced expression of *scl* that drives the formation of midtrunk endothelium^14^,^32^. To further validate that the effect of *clo* mutation was not due to a deficiency of unidentified non-endothelial gene(s), we utilized the *etv2* and *scl* morphants in our assays, while both of which were verified to show a loss of midtrunk vasculature (Fig. 6). The vascular phenotype of *etv2*MOs in this study was unlikely resulted from an off-targeting effect, as these MOs have been confirmed to phenocopy an ENU mutation of *etv2* in the early vasculogenesis^37^. Likewise, the endothelial phenotype of the *scl* morphant was consistent with that of a reported truncating mutation of *scl*^38^. Moreover, both the *clo* mutation and the knockdown of either *etv2* or *scl* were able to suppress migration defects of the head kidney, suggesting that the effect of *clo* on the endodermless embryo is most likely entitled to a loss of endothelium.

Our experiments using *sox32* mutant/morphant, *sox32/osr1* double morphants and a transplantation assay revealed that the presence of the endoderm is essential for the parallel migrations of the PCV and the head kidney. Apart from the well-known defects in endoderm and heart formation, the *sox32* morphant faithfully phenocopied the *sox32* mutant in terms of head kidney abnormalities (Figs 1 and 2). The loss of endoderm in the *sox32* morphant was verified by checking the *sox17* promoter-driven GFP fluorescence (Figs 4 and S8). Although Sox17 is expressed in arteries and required for arterial differentiation in mice^39^, the expression of *sox17* in the zebrafish is restricted to the endoderm and not detected in the vessels^40^,^41^. The association between endodermal cells and the PCV in the transplantation suggested that the PCV rather than the head kidney is directly influenced by the endodermal signalling (Fig. 4O–U). In addition, the grafted endodermal cells interacted preferentially with venous rather than arterial endothelium, which is consistent with our and other studies that morphology of venous instead of arterial vasculature was compromised in the endodermless embryo (Fig. 2)^23^.

Together with other studies, our results indicate a stepwise patterning of the developing kidney field by endothelium-derived signals. Prior to the DA-head kidney interplay^15^,^42^,^43^, the PCV interacts and co-migrates with head kidney cells during midline convergence. Moreover, the parallel migration of the head kidney with the PCV depends at least in part on VegfC-Flt4 signalling. This sequential regulation by the PCV and then the DA might therefore ensure the positioning of the head kidney at the radix of the PCV rostral to the DA-PCV joint (Fig. S1), while the paired pronephric tubules become juxtaposed to the PCV but not the DA by 2 dpf, a stage when both nephron assembly and definitive hematopoiesis occur. It is possible that differential patterns of paracrine signals or extracellular matrix microenvironments control region-specific kidney-vessel interactions during the nephron segmentation. Understanding the molecular and cellular mechanisms that govern such domain-specific interplays might help us to understand how the teleostean kidney is shaped to function in both blood filtration and definitive hematopoiesis.

Our results indicate that both the endoderm and the PCV are important for the early migration of the head kidney. However, head kidney cells actively converged toward the midline in the complete absence of endoderm and vasculature (Figs 5 and 6), supporting previous studies that have indicated that the motion of the bilateral mesoderm layers toward the midline is primarily due to the active migration of mesodermal cells^44^,^45^,^46^. Our results also suggest that the collective migration of head kidney cells requires endoderm only when the head kidney associates with nascent endothelial structures. It is thus intriguing to ask which mechanism drives the active migration of head kidney cells during the absence of endoderm and endothelium. Endodermal and mesodermal cells are co-regulated by paracrine signalling in a coordinated manner during midline convergence. Notably, noncanonical *wnt* signals play a role in the midline convergence of unpaired internal organs such as the heart, anterior gut tube, liver and pancreas^47^. It is unknown whether noncanonical *wnt* signalling regulates head kidney convergence either directly or indirectly. Nevertheless, our results suggest that the collective migration of head kidney cells, prior to the DA-head kidney interactions, could be subject to both (1) attractive signalling exerted by midline-derived paracrine factor(s) and (2) restrictive signalling by the PCV endothelium. In this scenario, it is likely that the PCV rather than the head kidney is directly influenced by the endoderm.

Our results also indicate that the venous niche of the early head kidney develops ahead of organ assembly and requires VegfC-Flt4 signalling. In mammals, VegfC is necessary for lymphangiogenesis in the kidney, and its down-regulation promotes the progression of polycystic kidney disease^48^. VegfC expressed in the kidney podocytes functions both as a paracrine factor to increase the stability and intracellular calcium of the glomerular endothelial monolayer^49^ and autonomously to promote survival in podocytes^50^. In contrast to these studies in mice, in which VegfC in the kidney podocytes directs the behaviour of glomerular and lymphangiogenic endothelial cells, our results suggest an early paracrine function of VegfC for the head kidney to interact with major venous vessels. It remains to be explored how VegfC/Flt4 signalling patterns the microenvironment at the interface of the head kidney and the PCV and how it influences the transport of HSC cells through the PCV to populate the peri-PCV sides of head kidney.

## Methods

### Ethics Statement

All experimental procedures on zebrafish were approved by the Institutional Animal Care and Use Committee of Tunghai University (IRB Approval NO. 101-12) and carried out in accordance with the approved guidelines.

### Zebrafish Husbandry

Zebrafish (*Danio rerio*) were reared according to standard protocols^51^. Embryos were obtained from natural crosses of wild-type, transgenic, or mutant fish and staged as previously described^52^. The following lines were used: *Tg(wt1b:GFP)(line 1)*^26^ (from Christoph Englert, Fritz-Lipmann Institute, Jena, Germany); *Tg(fli1:EGFP)*^*y1*^ (from Zebrafish International Research Center); *Tg(kdrl:mCherry)*^*ci5 *^53 (from Taiwan Zebrafish Core Facility)*; clo*^*m39*^ mutant^54^ and *Tg(kdrl:EGFP)*^*s843 *^23 (from Didier Stainier, Max Planck Institute for Heart and Lung Research, Bad Nauheim, Germany).

### 3β-Hsd Staining, ISH, IHC, Densitometry and Imaging

Embryos used for histological analysis were treated with 0.03% phenylthiourea (Sigma) from 12 h post-fertilization (hpf) onwards to inhibit pigmentation. 3β-Hsd activity staining, ISH^55^, and IHC^46^ were performed with modifications according to previously published methods.

To delineate the morphology of steroidogenic IR, histochemical staining for 3β-Hsd enzymatic activity was performed on whole embryos. Nomarski images of whole-mount or plastic-sectioned 3β-Hsd activity-stained embryos were captured using a BX51 microscope (Olympus).

To perform whole-mount ISH, digoxigenin-labelled riboprobes were synthesized from plasmids containing the *wt1a, wt1b, foxa2*, and *vegfC* genes. Fluorescein-labelled antisense riboprobes were synthesized from plasmids with the *ff1b (nr5a1a*) and *wt1b* genes. The probes were detected with alkaline phosphatase-conjugated anti-digoxigenin or anti-fluorescein antibodies (Roche) and visualized with 5-bromo-4-chloro-3-indolyl-phosphate/nitro blue tetrazolium (Promega) or Fast Red (Roche). Stained embryos were flat-mounted and photographed under a BX51 microscope (Olympus).

For IHC experiments on sectioned embryos, *Tg(wt1b:GFP)* embryos were fixed and embedded in 4% NuSieve GTG low-melting agarose (Lonza), cut into 100 μm sections with a VT1000M vibratome (Leica), and permeabilized with phosphate-buffered saline (PBS) containing 1% Triton X-100 before incubation with rabbit anti-human Fn (F3648, Sigma), anti-human VegfC (H-190, Santa Cruz), and mouse anti-chicken β-catenin (C7207, Sigma) antibodies at 1:200, 1:50 and 1:50, respectively. The whole-mount IHC experiment using the α6F monoclonal antibody (DSHB) at 1:25 was performed as described by Drummond *et al*.^8^ Dylight 594- and 650-conjugated anti-rabbit or anti-mouse IgG (Abcam) were used as secondary antibodies at 1:200. Images were captured with an LSM510 confocal microscope with version 3.5 software (Zeiss).

To quantify the distances between various tissue structures, images of embryos in each group were taken with identical magnification using an Axioskop 2 Plus microscope equipped with AxioVision 3.0 software (Carl Zeiss).

### Microinjection of Antisense MO Oligonucleotides

MO oligonucleotides were synthesized at Genetools, LLC. The nucleotide sequences of the MOs were: *sox32*MO, 5′-CAG GGA GCA TCC GGT CGA GAT ACA T-3′^56^; *pkd2*MO, 5′-AGG ACG AAC GCG ACT GGA GCT CAT C-3′^57^; *osr1*MO, 5′-ATC TCA TCC TTA CCT GTG GTC TCT C-3′^30^; *etv2*MO1, 5′-TTG GTA CAT TTC CAT ATC TTA AAG T-3′^33^; *etv2*MO2, 5′-CAC TGA GTC CTT ATT TCA CTA TAT C-3′^33^; *scl* E1/IMO, 5′-GCG GCG TTA CCT GTT AAT AGT GGC G-3′^58^; *scl* E2/IMO, 5′-AAT GCT CTT ACC ATC GTT GAT TTC-3′^58^; *vegfC*MO, 5′-GAA AAT CCA AAT AAG TGC ATT TTA G-3′^6^; *flt4*MO, 5′-TTA GGA AAA TGC GTT CTC ACC TGA G-3′^59^; STD-MO, 5′-CCT CTT ACC TCA GTT ACA ATT TAT A-3′. A 2 mM stock solution was prepared by dissolving lyophilized MO powder in 1 x Danieau solution before further dilution into the required concentrations prior to injection into one- to two-cell stage embryos using a Nanoject (Drummond Scientific Company). *sox32*MO, *pkd2*MO, *osr1*MO, and STD-MO were injected at dosages of 0.5, 0.5, 0.9 and 1.4 pmole per embryo, respectively. *scl*MO represents an equimolar mixture of *scl*E/IMO and *scl*E2/IMO at a dosage of 1.2 pmole injected per embryo, and *etv2*MO represents an equimolar mixture of *etv2*MO1 and *etv2*MO2 at a dosage of 1.2 pmole injected per embryo.

### Transplantation

Transplantations were performed using methods similar to those described in ref. 60. Briefly, the wild-type AB strain was used as a donor. Eggs used for donor embryos were labelled at the 2- to 4-cell stage by micro-injection with a mixture of fluorescein dextran (Alexa Fluor fixable 568, 10,000 MW, from Invitrogen; 5% of dextran dye in 0.2 M KCl) and *sox32* mRNA (20 ng/ul). Eggs used for the recipients were microinjected with *sox32* MO (5 ng/ul) at the one-cell stage. Between 20–40 cells were then taken directly from the animal pole of a labelled donor embryo at the midblastula stage (approximately 1,000–2,000 cells) and transplanted along the blastoderm margin of the recipients at approximately 4 hpf without damaging the yolk of the MO-injected host embryos. Host embryos were allowed to develop further for microscopy and photography using an LSM510 confocal microscope with version 3.5 software (Zeiss).

### Statistical Analysis

All quantitative data are expressed as the mean ± standard error of the mean. Data were evaluated by analysis of variance (ANOVA) followed by Duncan’s new multiple range test (Duncan’s multiple test) or Student’s t test. *P* < 0.05 was considered statistically significant.

## Additional Information

**How to cite this article**: Chou, C.-W. *et al*. The endoderm indirectly influences morphogenetic movements of the zebrafish head kidney through the posterior cardinal vein and VegfC. *Sci. Rep.* **6**, 30677; doi: 10.1038/srep30677 (2016).

## Supplementary Material

Supplementary Information
