# Supplementary material for: The endoderm indirectly influences morphogenetic movements of the zebrafish head kidney through the posterior cardinal vein and VegfC
Source: Sci Rep. 2016 Aug 1;6:30677. doi: 10.1038/srep30677 (PMC4967926; doi:10.1038/srep30677)
Supplement: Supplementary Information [file srep30677-s1.pdf]

SUPPLEMENTARY INFORMATION

The endoderm indirectly influences morphogenetic movements of the zebrafish head kidney through the posterior cardinal vein and VegfC

Chih-Wei Chou, Hsiao-Chu Hsu, May-su You, Jamie Lin and Yi-Wen Liu*

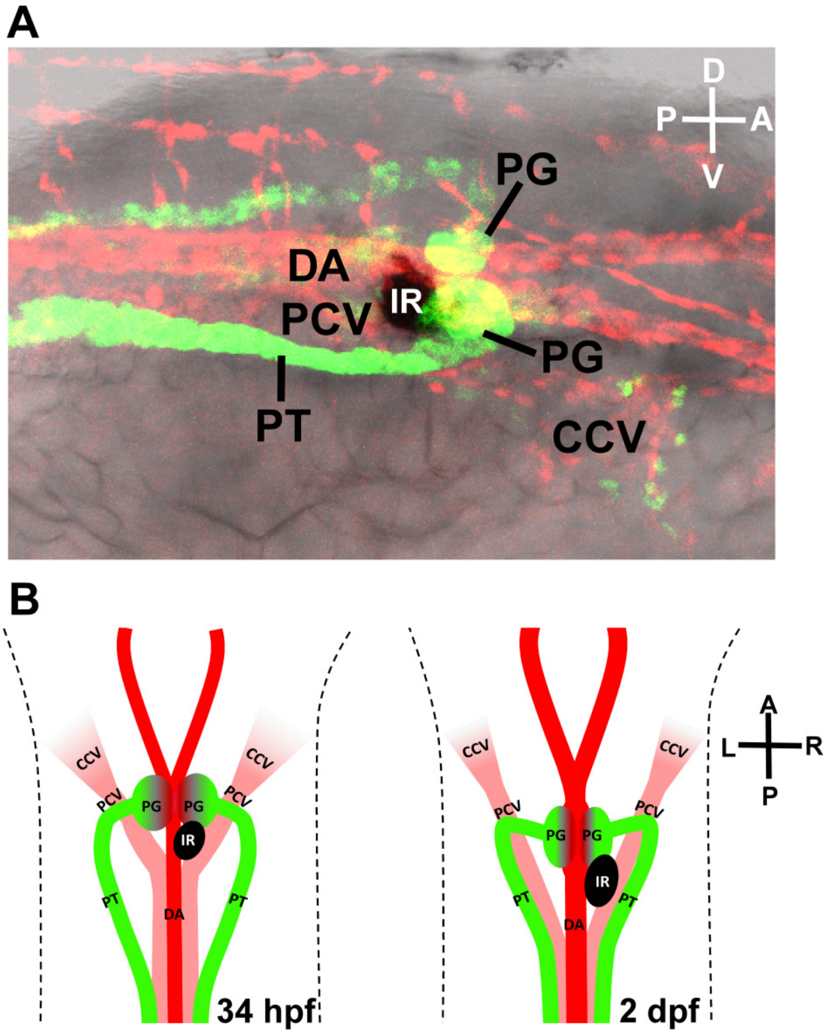

Figure S1. The relative spatial distribution of the PG, the IR, the DA, the PCV, the pronephric tubules (PT), and the CCV is shown by (A) a dorsolateral view of the 3 β -Hsd activity stained *Tg(wt1b:GFP)*; *Tg(kdrl:mCherry)*^{ci5} embryo oriented with anterior to the right and (B) a schematic depicting the dorsal view of head kidney structures and axial vessels at the stage of 34 hpf and 2 dpf respectively. D, dorsal; V, ventral; A, anterior; P, posterior; R, right; L, left.

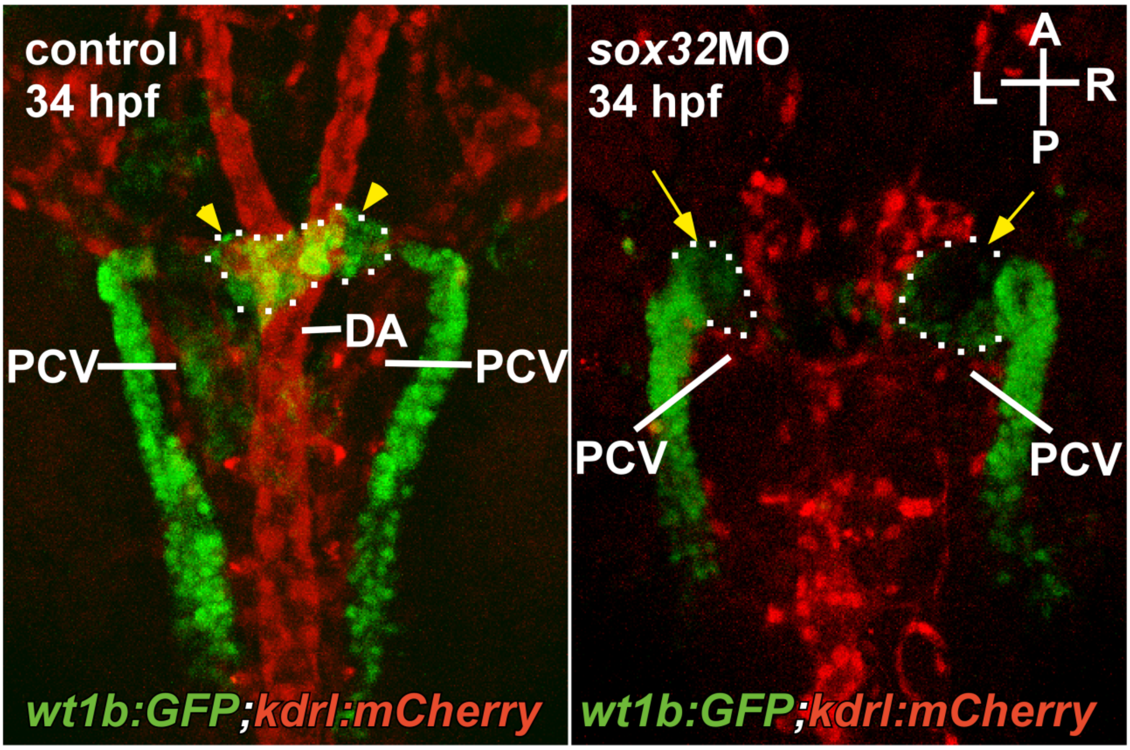

Figure S2. Angiogenesis of the PG was defective in the *sox32* morphant. The pronephric kidney and the blood vessels are delineated by green and red fluorescence respectively in the *Tg(wt1b:GFP)*;*Tg(kdrl:mCherry)^{ci5}* embryo at the stage of 34 hpf. The relative spatial distribution of the PG (yellow arrowheads), the DA and the PCV is shown in a dorsal view. Yellow arrows indicate defective angiogenesis of the PG. A, anterior; P, posterior; R, right; L, left.

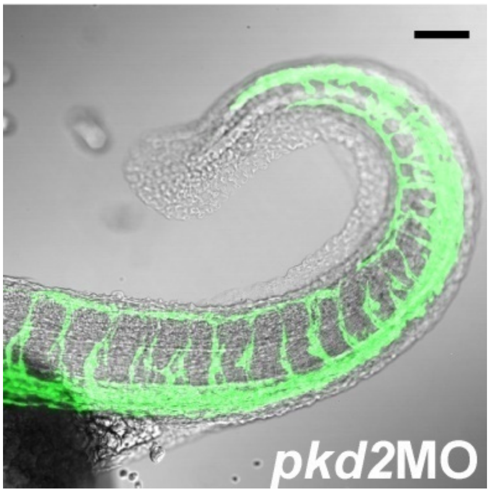

Figure S3. The curly up tail phenotype of the *Tg(fli1:EGFP)*^{y1} embryo injected with the *pkd2* morpholino. Scale bar, 50 μm.

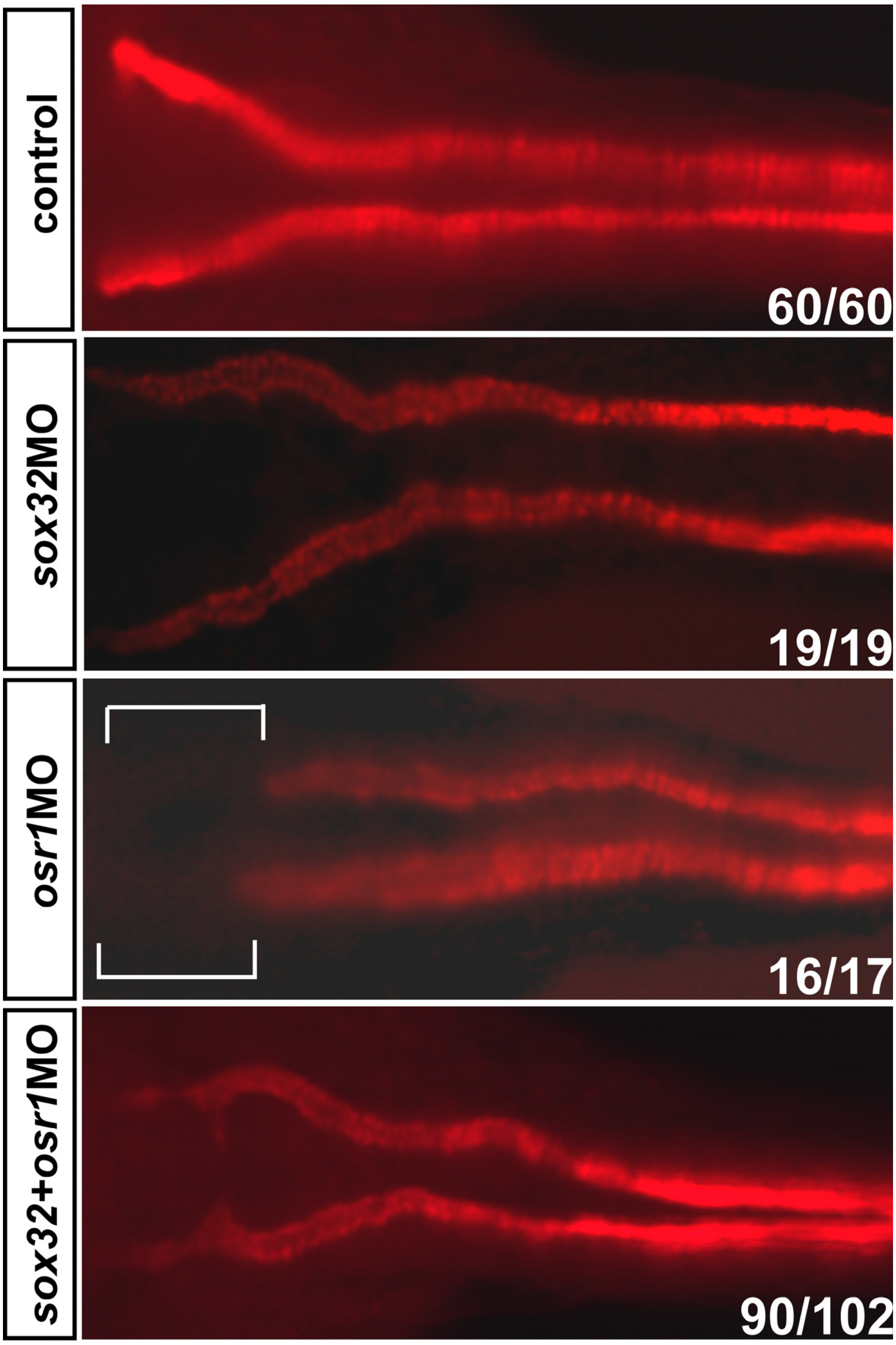

Figure S4. Compromised formation of proximal convoluted tubules at 53 hpf in the *osr1* morphant was rescued upon a co-injection of *sox32*MO. The pronephric tubules were labelled by the α6F antibody that detects Na^+/K^+ATPase. The bracketed region in the *osr1* morphant denotes a loss of proximal convoluted tubules.

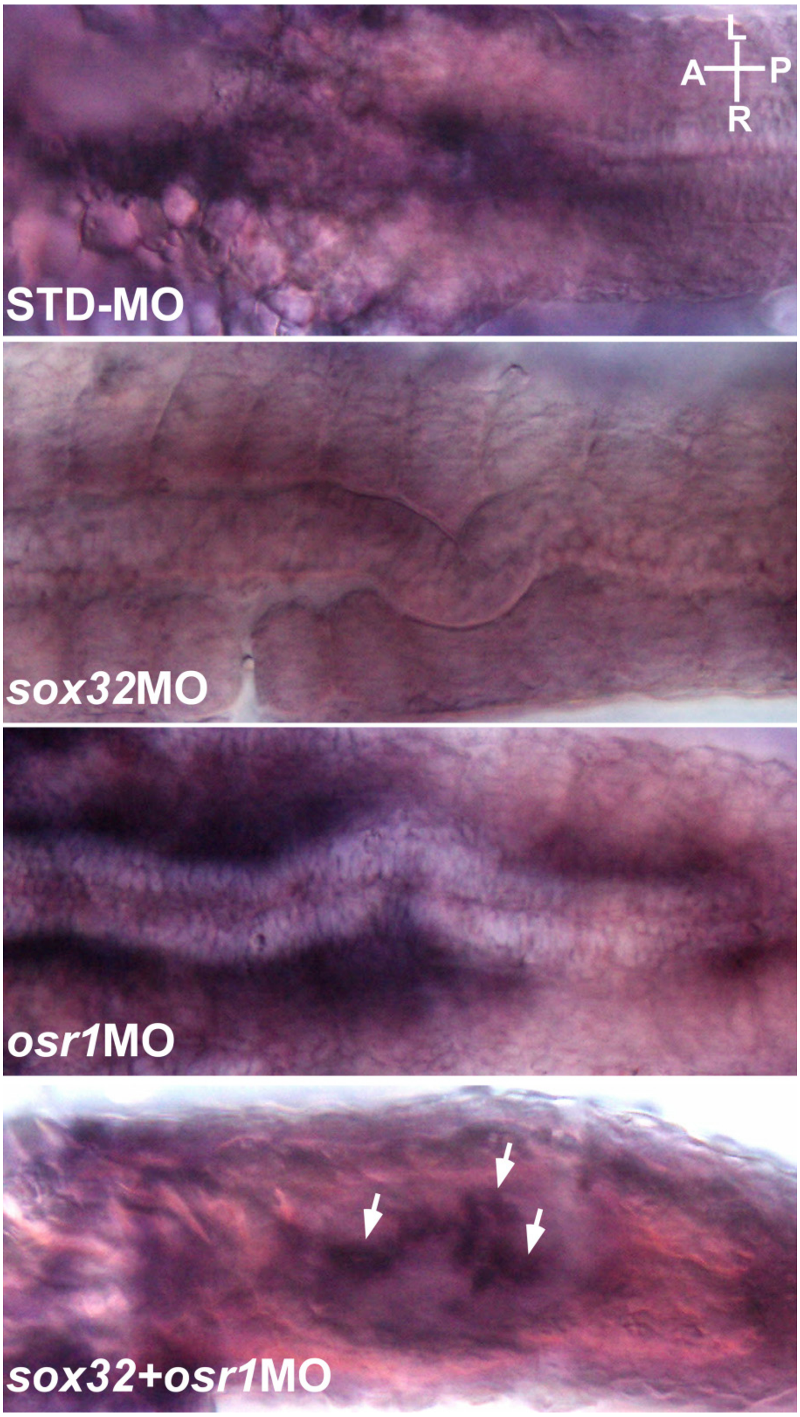

Figure S5. Ventral views of the embryos injected with STD-MO, *sox32*MO, *osr1*MO and *sox32*/*osr1*MO, respectively, and analysed by ISH for detecting the *foxa2* expression at the midtrunk at 24 hpf. White arrows indicate the rescued *foxa2* expression in the *sox32/osr1* double morphant. The images shown are representative of 19, 15, 17 and 23 samples respectively. A, anterior; P, posterior; R, right; L, left.

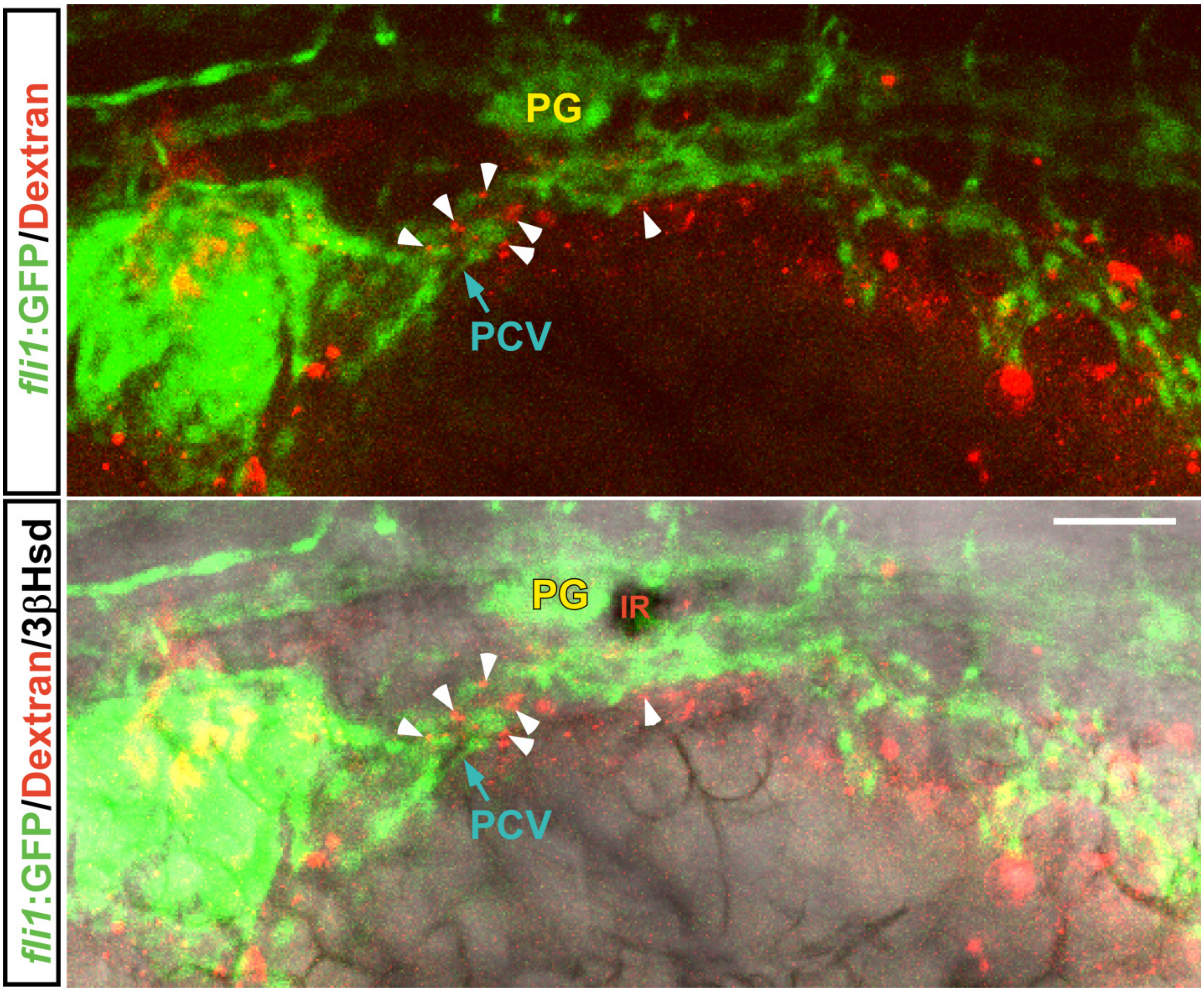

Figure S6. A magnified dorsolateral view of the transplanted embryo shown in Figure 4P. Grafted *sox32*RNA⁺Dextran⁺ cells (marked by white arrowheads) were associated with the PCV endothelium in the *sox32*MO-injected host embryo.

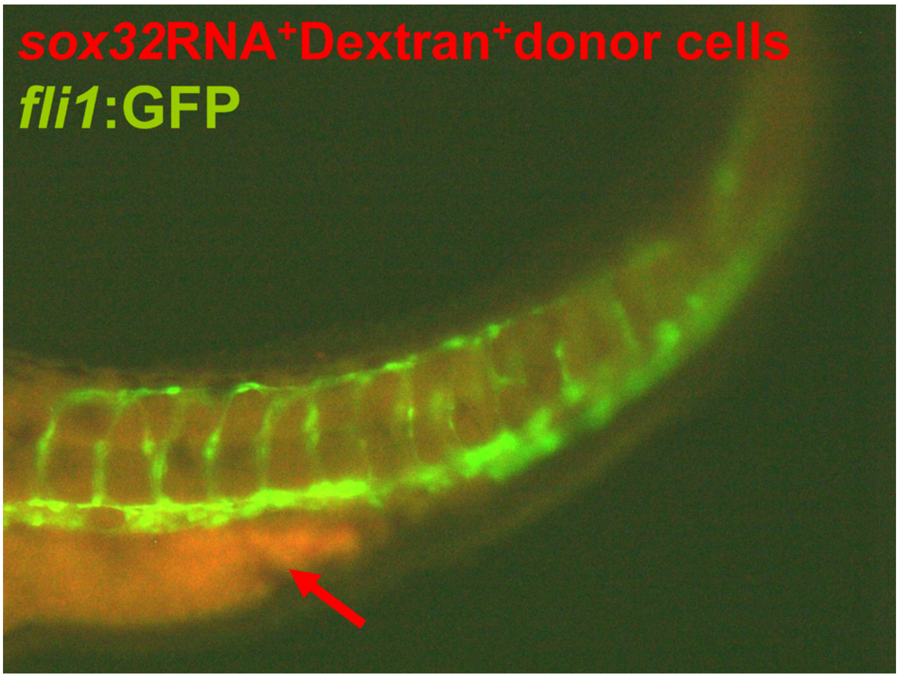

Figure S7. The donor cells from the embryo injected with *sox32* mRNA and fluorescent dextran developed into a gut tube-like structure at the posterior trunk of the *sox32MO*-injected host embryo.

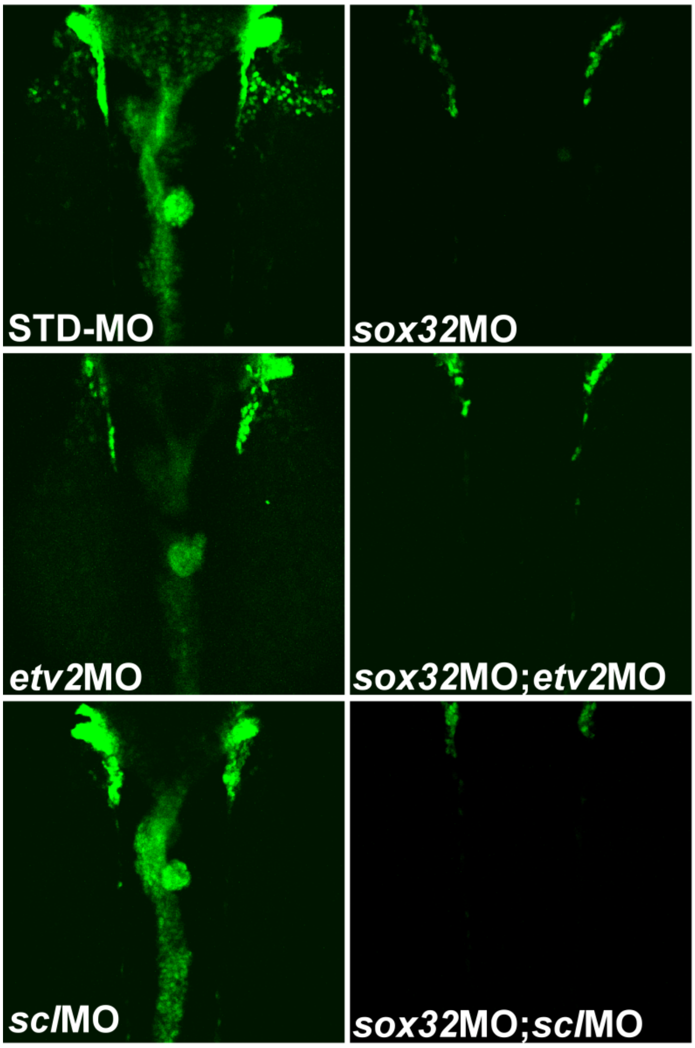

Figure S8. The endoderm formation delineated by the fluorescence of *Tg(sox17:EGFP)*^{s870} was inhibited by the *sox32*MO, which was injected individually or co-injected with either *etv2*MO or *scl*MO.

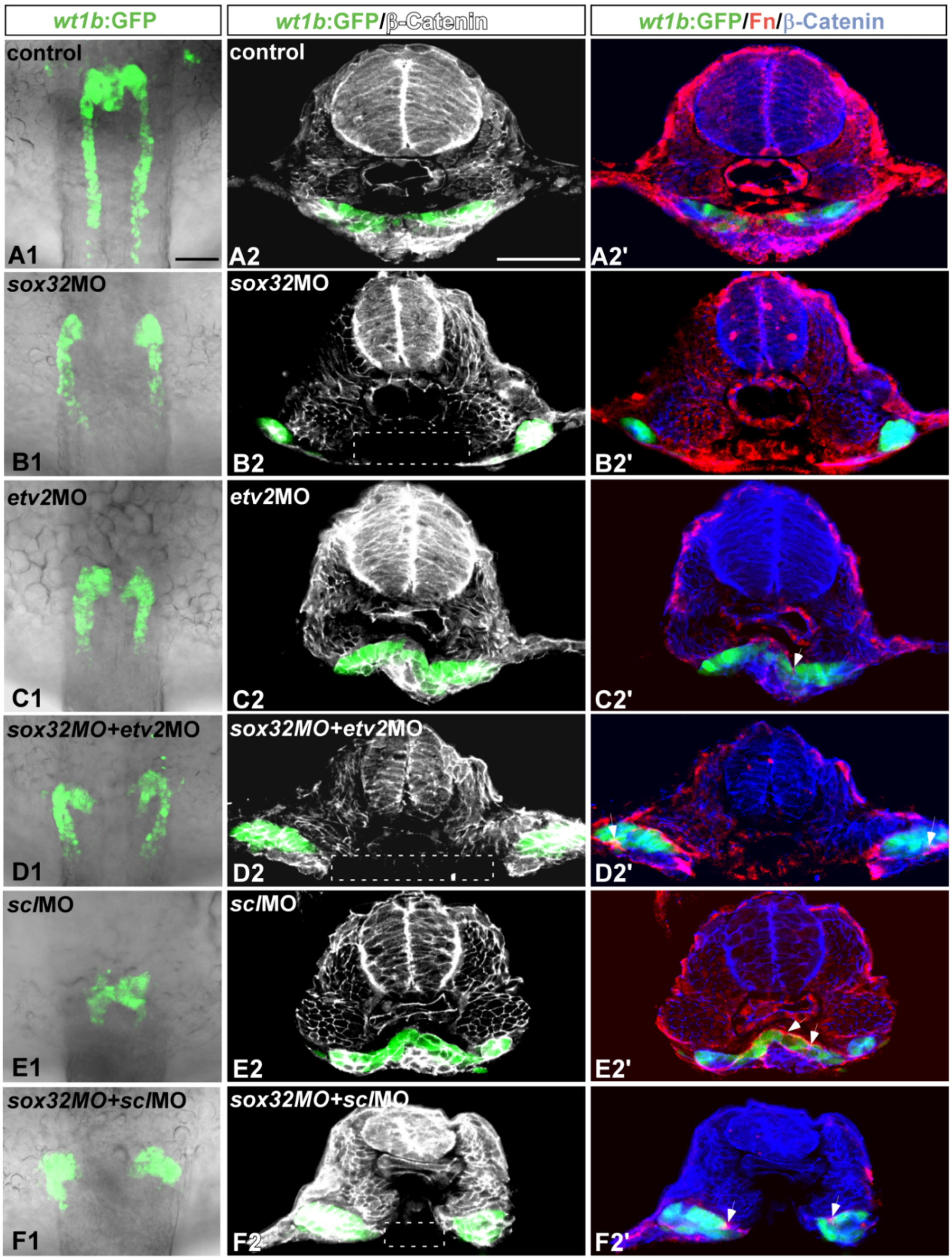
wt1b:GFP
wt1b:GFP/β-Catenin
wt1b:GFP/Fn/β-Catenin
control
A1
A2
A2'
sox32MO
B1
B2
B2'
etv2MO
C1
C2
C2'
sox32MO+etv2MO
D1
D2
D2'
scl/MO
E1
E2
E2'
sox32MO+scl/MO
F1
F2
F2'

Figure S9. The defective midline convergence of the kidney in the *sox32* morphant prior to interrenal differentiation was not suppressed by either *etv2*MO or *scl*MO. *sox32*MO, *etv2*MO, *scl*MO, *sox32/etv2* double-MOs, *sox32/scl* double-MOs and STD-MO were injected into *Tg(wt1b:GFP)* embryos to test their effects on kidney morphology at 24 hpf. (A1-F1) The kidney morphology was delineated by the expression of *wt1b:GFP* in the dorsal whole-mount views of the midtrunk. (A2-F2) Cross-sections showing the expression of *wt1b:GFP* and β-Catenin (white). (A2'-F2') Cross-sections showing the expression of *wt1b:GFP*, Fn (red) and β-Catenin (blue). The loss of β-Catenin in the ventral region is highlighted by white rectangles, and Fn deposits are marked by white arrows. The images shown are representative of 3, 15, 20, 16, 20 and 11 samples for control embryos, *sox32* morphants, *etv2* morphants, *etv2/sox32* double morphants, *scl* morphants and *scl/sox32* double morphants, respectively. Scale bar, 50 μm.

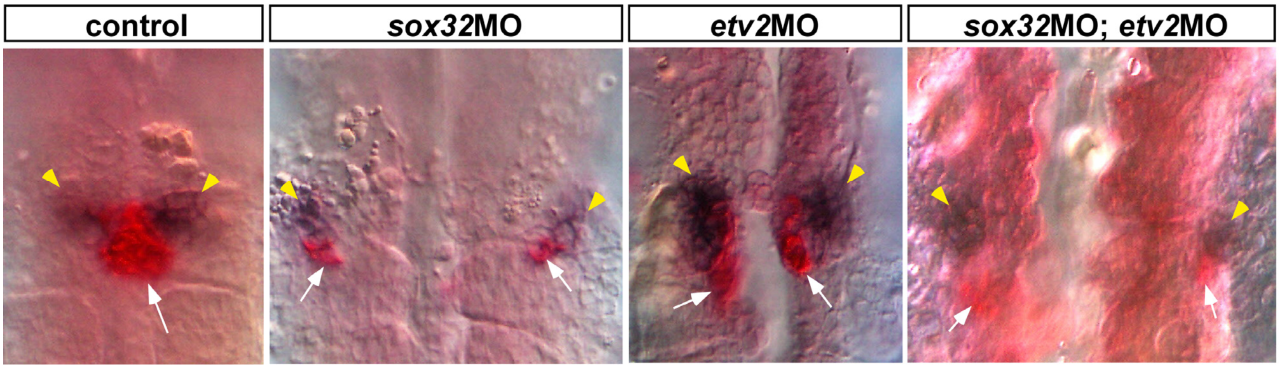

Figure S10. Expression patterns of *ff1b* (red) and *wt1b* (dark purple) were detected by double ISH in the *sox32* morphant, the *etv2* morphant, the *sox32/etv2* double morphant and the control embryo at 24 hpf. Localizations of the PG (marked by yellow arrowheads) and the IR (marked by white arrows) are viewed ventrally.
